# Strengthening epidemiological surveillance in primary healthcare to combat Hansen’s disease: reflections on the disease in the Brazilian context, 2025

**DOI:** 10.1590/S2237-96222025v34e20240019.en

**Published:** 2025-08-04

**Authors:** Guilherme Almeida Elidio, Dirce Bellezi Guilhem

**Affiliations:** 1 Universidade de Brasília, Faculdade de Ciências da Saúde, Brasília, DF, Brazil

**Keywords:** Hansen’s disease, Epidemiologic Surveillance Services, Health Centers, Primary Health Care, Epidemiology

## Abstract

Not applicable.

The study conducted by Barros (1) provides robust and detailed data that warn about the Hansen’s’s Disease (leprosy) scenario in Brazil and the fulfillment of the Ministry of Health’s strategic plan to combat Hansen’s disease in the period 2024-2030. The study found that, in fact, there was a reduction in the numbers, proportions and detection rates of the disease in the state of Piauí (1). However, when considering the specificities and complexity of Hansen’s disease, these results deserve some reflection.

The average annual detection rate of new cases of Hansen’s disease in Piauí was 36.5/100,000 inhabitants. In Brazil as a whole, this rate was 9.67/100,000 inhabitants, being higher for new cases of multibacillary Hansen’s disease (22.5/100,000 inhabitants). This finding represented risk of the endemicity of the diease being maintained, since multibacillary cases constitute the group that disseminates the disease, if not treated (2).

According to the results obtained by Barros (1), despite there being a decreasing trend in the detection of new cases of Hansen’s disease in children under 15 years of age, this is still 10.7/100,000 inhabitants. This rate is striking, considering the World Health Organization’s precepts for combating the disease, as it establishes that the existence of new cases of Hansen’s disease in people under 15 years of age indicates continuity of the transmission chain (3).

When considering the findings of Barros (1), in which the incidence rates of new cases of Hansen’s disease are present, this could represent a major challenge for public health, not only in Piauí, but throughout the country, causing Brazil to move away from its mission of certifying itself as “A Brazil free from Hansen’s disease”.

Globally, the main strategies for tackling Hansen’s disease are implementing basic research on the disease, ensuring the supply of drugs, tackling stigma and discrimination, and maintaining the capacity to detect new cases in Primary Health Care (4). 

One of the first strategies to be considered in this context is to understand that there is no timely and immediate response to Hansen’s disease if epidemiological surveillance continues without integration of primary health care centers, where identification, testing and treatment are provided to these patients. 

In order to combat Hansen’s disease, health managers could establish, epidemiology centers in Primary Health Care with professionals responsible for the investigation, treatment and outcome of this disease. Without a doubt, this initiative would provide Brazil with the non-endemic status recommended recommended by the World Health Organization.
