# Supplementary material for: Strengthening epidemiological surveillance in primary healthcare to combat Hansen’s disease: reflections on the disease in the Brazilian context, 2025
Source: Epidemiol Serv Saude. 2025 Aug 4;34:e20240019. doi: 10.1590/S2237-96222025v34e20240019.en (PMC12338986; doi:10.1590/S2237-96222025v34e20240019.en)
Supplement: Translation

# Translation: O reforço da vigilância epidemiológica na atenção primária à saúde no combate à hanseníase: reflexões da doença no contexto brasileiro, 2025

**DOI:** 10.1590/S2237-96222025v34e20240019.pt

**Authors:** Guilherme Almeida Elidio, Dirce Bellezi Guilhem

**Affiliations:** 1 Universidade de Brasília, Faculdade de Ciências da Saúde, Brasília, DF, Brasil

**Keywords:** Hanseníase, Serviços de Vigilância Epidemiológica, Centros de Saúde, Atenção Primária à Saúde, Epidemiologia

## Abstract

Não se aplica

O estudo de Barros (1) traz dados robustos e ricos em detalhes que alertam para o cenário da hanseníase no Brasil e o cumprimento do plano estratégico do Ministério da Saúde para enfrentamento à hanseníase no período 2024-2030. No estudo, verifica-se que, de fato, houve redução dos números, das proporções e das taxas de detecção da doença no Piauí (1). No entanto, se considerarem as especificidades e a complexidade da hanseníase, é relevante fazer algumas reflexões sobre esses resultados.

A taxa média de detecção anual de casos novos de hanseníase no Piauí foi de 36,5/100 mil habitantes. No Brasil, essa taxa foi de 9,67/100 mil habitantes, sendo superior para casos novos de hanseníase multibacilar (22,5/100 mil habitantes). Esse achado representou risco de manutenção da endemicidade da doença, visto que os casos multibacilares constituem o grupo disseminador da doença, caso não estejam sendo tratados (2).

Segundo os resultados de Barros (1), apesar de haver tendência decrescente para detecção de novos casos de hanseníase em menores de 15 anos de idade, essa taxa ainda é de 10,7/100 mil habitantes. Esse índice chama a atenção, considerando-se os preceitos da Organização Mundial da Saúde (OMS) no combate à doença, a qual estabelece que a existência de casos novos de hanseníase em pessoas com menos de 15 anos indica continuidade da cadeia de transmissão (3).

Se considerarem os achados de Barros (1), estudo no qual as taxas de incidência de novos casos de hanseníase estão presentes, pode significar um grande desafio para a saúde pública, não apenas do Piauí, mas sim de todo o país, que pode se distanciar da missão de certificar-se como “Um Brasil sem hanseníase”.

Globalmente, as principais estratégias para o enfrentamento da hanseníase são a implementação de pesquisas básicas sobre a doença, a garantia do fornecimento de medicamentos, o enfretamento ao estigma e à discriminação e a manutenção da capacidade de detectar casos novos de hanseníase na Atenção Primparia à Saúde (APS) (4).

Uma das primeiras estratégias a ser considerada nesse contexto é compreender que não há resposta oportuna e imediata à hanseníase, caso se mantenha a vigilância epidemiológica desintegrada das unidades básicas de saúde, lugar onde se faz a identificação, testagem e oferta de tratamento a esses pacientes. 

No combate à hanseníase, os gestores de saúde poderiam instituir, nos serviços de APS, núcleos de epidemiologia com profissionais responsáveis pelo processo de investigação, tratamento e desfecho dessa doença. Sem dúvidas, essa iniciativa traria ao Brasil o status de situação não endêmica preconizado pela OMS.
